# Association of COVID‐19 Continuous Enrollment With Self‐Reported Postpartum Medicaid Continuity and Coverage Inequities

**DOI:** 10.1111/1475-6773.14618

**Published:** 2025-03-26

**Authors:** Erica L. Eliason, Sarah H. Gordon, Maria W. Steenland

**Affiliations:** ^1^ Center for State Health Policy, Rutgers University New Brunswick New Jersey USA; ^2^ Department of Urban‐Global Public Health Rutgers School of Public Health Newark New Jersey USA; ^3^ Department of Health Law, Policy & Management Boston University School of Public Health Boston Massachusetts USA; ^4^ Population Studies and Training Center, Brown University Providence Rhode Island USA; ^5^ Department of Family Science University of Maryland School of Public Health College Park Maryland USA

**Keywords:** COVID‐19, health equity, health insurance, Medicaid, medically uninsured, postpartum period

## Abstract

**Objective:**

To examine the impact of extended postpartum Medicaid eligibility under the Families First Coronavirus Response Act (FFCRA) on self‐reported postpartum insurance status among prenatal Medicaid recipients, and differences by state Medicaid expansion status and race, and ethnicity.

**Study Setting and Design:**

We used a global polynomial linear regression discontinuity design (RDD) approach to estimate the effect of extended postpartum Medicaid eligibility during the FFCRA on changes in self‐reported postpartum Medicaid, private coverage, and uninsurance. This approach compares individuals who gave birth before FFCRA exposure with those who gave birth during extended postpartum Medicaid eligibility, using birth timing to determine FFCRA exposure. We estimated RDD models overall, by state Medicaid expansion status, and by race and ethnicity.

**Data Sources and Analytic Sample:**

This study used 2018–2021 Pregnancy Risk Assessment Monitoring System data, a multi‐state survey of individuals with a recent live birth, and a sample of prenatal Medicaid recipients age 20 or older in 29 study jurisdictions.

**Principal Findings:**

In adjusted RDD models, extended Medicaid eligibility was associated with a 10.7 percentage point (pp) (95% CI: 8.7, 12.6) increase in postpartum Medicaid, a 3.5 pp (95% CI: −5.2, −1.8) decrease in postpartum private coverage, and a 6.5 pp (95% CI: −8.0, −5.0) decrease in postpartum uninsurance. In stratified RDD models, we found larger increases in postpartum Medicaid and larger decreases in uninsurance in non‐expansion states than in Medicaid expansion states. In RDD models by race and ethnicity, we found similar increases in postpartum Medicaid and similar decreases in postpartum uninsurance among non‐Hispanic Black respondents, Hispanic respondents, and non‐Hispanic White respondents.

**Conclusions:**

We found significant improvements in postpartum Medicaid continuity and reductions in uninsurance during extended postpartum Medicaid eligibility. Postpartum Medicaid extensions under the American Rescue Plan could help maintain some coverage gains under the FFCRA.

**Summary**

- What is known on this topic
  ○ Previous research found improvements in postpartum Medicaid continuity under extended Medicaid eligibility during the Families First Coronavirus Response Act (FFCRA), with mixed findings on transitions to private coverage and uninsurance.
  ○ Extended Medicaid eligibility could be particularly important for improving coverage in non‐expansion states and among Hispanic postpartum people, who had high rates of Medicaid loss prior to the FFCRA.
  ○ The FFCRA extended Medicaid eligibility is of particular relevance in the postpartum period due to 12‐month postpartum Medicaid extensions adopted in almost all states under the American Rescue Plan Act.
- What this study adds
  ○ Extended Medicaid eligibility was associated with significant improvements in self‐reported postpartum Medicaid continuity among prenatal Medicaid enrollees overall, with declines in postpartum private coverage and uninsurance.
  ○ In stratified models, postpartum Medicaid increases were larger in non‐expansion states than in Medicaid expansion states; similar increases in postpartum Medicaid were observed among Hispanic, non‐Hispanic Black, and non‐Hispanic White respondents.
  ○ State 12‐month postpartum Medicaid extensions could help maintain some gains in postpartum Medicaid; however, additional efforts may be needed to address racial–ethnic inequities as high uninsurance persisted among Hispanic respondents.

## 1 Introduction

The United States is experiencing a maternal health crisis, marked by rising rates of maternal mortality and stark racial and ethnic health inequities [1, 2]. Rates of pregnancy‐related mortality among non‐Hispanic Black birthing people are three times higher than among non‐Hispanic White birthing people [1]. Medicaid covers four in 10 births in the US and over half of births among Black and Hispanic birthing people [3]. Pregnancy‐related Medicaid is available for pregnant people with incomes up to 138% of the federal poverty level (FPL) in all states, but eligibility in most states greatly exceeds the minimum (median across states: 205% FPL in 2020). In 2020, median income eligibility for Medicaid as an adult/parent was 138% FPL in states that expanded Medicaid and 41% FPL in states that did not, creating an eligibility gap between pregnancy and other eligibility pathways [4]. Federal law mandates that pregnancy‐related Medicaid must be provided through the end of the month in which 60 days postpartum occurs. After this period, individuals must qualify through another pathway, such as parental Medicaid, to retain Medicaid. Otherwise, pregnancy‐related Medicaid recipients must transition to another insurance in the postpartum period or risk becoming uninsured. This has led to higher rates of Medicaid enrollment during pregnancy compared to after the end of the pregnancy‐related Medicaid enrollment period [5].

By increasing Medicaid eligibility for non‐pregnant adults, Medicaid expansion under the Affordable Care Act (ACA) increased continuous postpartum Medicaid [6, 7]. However, only 68% of prenatal Medicaid enrollees reported consistent Medicaid coverage through 9–10 months postpartum in 2019, after most states had expanded Medicaid [8]. In addition, racial and ethnic disparities persisted among individuals with Medicaid‐paid prenatal care in 2015–18, with the highest postpartum Medicaid loss among Hispanic birthing parents [5]. Increasing postpartum Medicaid continuity is the primary strategy recommended by the federal government to address the maternal health crisis and racial–ethnic maternal health inequities [9, 10].

The start of the COVID‐19 public health emergency (PHE) in the US brought important changes to Medicaid, with the potential to impact postpartum Medicaid continuity. Under the Families First Coronavirus Response Act (FFCRA), states could receive enhanced federal matching funds in exchange for the continuous enrollment of Medicaid recipients regardless of fluctuations in eligibility [11]. As all states opted in, this created a de facto extension of Medicaid nationally beyond 60 days postpartum from March 2020 until April 2023. After April 2023, most states continued to provide a year of continuous postpartum Medicaid using a provision of the 2021 American Rescue Plan Act (ARPA) that gave states the option to extend pregnancy‐related Medicaid from 60 days to 12 months postpartum [12]. As of January 2025, 48 states and the District of Columbia (DC) have adopted these 12‐month postpartum Medicaid extensions [12]. Evidence from the continuous enrollment provision can offer early insight into the potential impact from present‐day policies extending Medicaid through 12 months postpartum.

Previous research found improvements in consistent postpartum Medicaid during the PHE, with mixed findings on transitions to private coverage and uninsurance [13, 14, 15, 16, 17]. One study using Medicaid claims found continuous Medicaid through the postpartum year increased from 59.3% before the pandemic to 90.7% during the pandemic [15]. However, research using a continuous difference‐in‐differences (DID) research design and postpartum survey data found a much more modest increase in continuous Medicaid, with no statistically significant change in uninsurance [16]. These findings are consistent with evidence that millions of Medicaid enrollees were unaware of continued enrollment and reported being uninsured in survey data (the “Medicaid undercount”) [18]. Additionally, some immigrant groups who received public insurance were not eligible for FFCRA continuous enrollment [19].

While previous analyses used Medicaid claims to examine changes in the duration of postpartum Medicaid, use of Medicaid claims does not provide information about changes in overall health insurance (i.e., including either a private or public payer) [15]. Use of survey data allowed us to examine whether the policy affected postpartum insurance as well as transitions in coverage type, such as from private coverage or uninsurance. Previous analysis of postpartum survey data identified the effects of a 100% FPL increase in postpartum Medicaid eligibility during the PHE with postpartum outcomes [16]. As the actual increase in eligibility varies by state, these results provide a hypothetical estimate of the impact of increasing eligibility by 100% in an average state. Our regression discontinuity design (RDD) approach provides an overall estimate of the observed association of extended Medicaid eligibility with postpartum coverage among all states in the analysis. Additionally, our approach also allowed for stratification by Medicaid expansion status, an analysis that would be challenging using the continuous DID, since most states with a large increase in eligibility were non‐expansion states.

In this study, we used a novel RDD approach to examine the impact of extended Medicaid eligibility on (i) self‐reported postpartum insurance among prenatal Medicaid recipients, and assess differences by (ii) state Medicaid expansion status and (iii) race and ethnicity for non‐Hispanic Black, Hispanic, and non‐Hispanic White respondents. We also compared the characteristics of prenatal Medicaid recipients who reported postpartum uninsurance with those who reported Medicaid. Evidence from this study could help policymakers anticipate the effects of state 12‐month Medicaid extensions on postpartum insurance, enrollment awareness, and coverage inequities.

## 2 Methods

### 2.1 Data and Sample

This study used 2018–2021 Pregnancy Risk Assessment Monitoring System (PRAMS) data, a multi‐state survey of individuals with a recent live birth sampled 2–6 months after childbirth from birth certificate files in participating states [20]. PRAMS is a state‐based surveillance project implemented by health departments in participating jurisdictions in collaboration with the Centers for Disease Control and Prevention (CDC) [20]. Although nearly all states participated in PRAMS, the CDC does not release data from jurisdictions that did not meet the 50% response rate criteria for each year. To ensure that inconsistent state inclusion was not driving changes in outcomes over time, we only included states that met the response rate criteria in all study years. This approach resulted in a sample of 22 states and New York City, with an annual median weighted response rate ranging from 56.4% to 60.5% (Methods S1). To focus on individuals eligible for extended postpartum Medicaid, we limited our sample to respondents who reported Medicaid‐paid prenatal care. We restricted to individuals ages 20 and older as adolescents are eligible for public insurance from the Children's Health Insurance Program (CHIP), which has higher income eligibility outside of pregnancy [21]. This study was considered not human subjects research by the Rutgers University institutional review board.

### 2.2 Variables

Our outcomes were postpartum health insurance type, self‐reported at the time of the survey (mean: 4 months postpartum). Postpartum coverage was categorized as Medicaid, private or military coverage, dual Medicaid and private or military coverage, and uninsurance, which included individuals with no coverage or only Indian Health Service coverage [22]. Respondents could select more than one insurance type, and for our main outcomes, individuals could report having multiple coverage types. We created mutually exclusive insurance categories as a sensitivity analysis.

We examined several demographic characteristics using PRAMS and linked birth certificate data, including age at delivery (20–24, 25–29, 30–35, 35 or older), marital status (married, unmarried), educational attainment (high school or less, some college, 4+ years of college), urban or rural residence, parity (primiparous, multiparous), race and ethnicity (non‐Hispanic Asian or Pacific Islander, non‐Hispanic Black, Hispanic, non‐Hispanic Indigenous [American Indian or Alaskan Native], non‐Hispanic White, non‐Hispanic other [mixed race or other race/ethnicity]), interview language (English, Spanish, Chinese), and whether a respondent had a preconception health condition (preconception type 1 or type 2 diabetes, high blood pressure or hypertension, or depression). We considered race and ethnicity due to the systematic and persistent racial inequities in health care resulting from institutional and structural racism and discrimination. We included indicators for missing data in any demographic variables.

### 2.3 Study Design

We used a RDD to estimate the effect of extended postpartum Medicaid eligibility during the FFCRA, which went into effect on March 18, 2020, by comparing individuals who gave birth prior to extended Medicaid eligibility (January 2018–December 2019) with individuals who gave birth during extended Medicaid eligibility (January 2020–December 2021). This study design assumes that individuals who gave birth just before and just after the cutoff were similar, and that differences in postpartum coverage can therefore be attributable to differences in exposure to extended Medicaid eligibility. Although the FFCRA occurred concurrently with the COVID‐19 pandemic, the continuous enrollment provisions were the major change that could affect Medicaid coverage; although some birthing people may have transitioned to Medicaid if they lost employer‐sponsored insurance [23]. We tested for discontinuities in characteristics to confirm the comparability of the population who gave birth before and after the January 2020 cutoff for the RDD approach.

This RDD‐in‐time framework uses time as the running variable, considering treatment of extended Medicaid eligibility past 60 days postpartum to begin at a cutoff of January 2020. We selected January 2020 as the policy exposure start date because some individuals with births in January 2020 would have otherwise lost pregnancy coverage in the absence of FFCRA in March 2020, and were therefore the first birth cohort with extended eligibility available to them due to the continuous enrollment policy.

### 2.4 Statistical Analysis

In our primary specifications, we used global polynomial linear regression models for the RDD. Although we considered higher‐order polynomials using quadratic and cubic terms, these were not selected as these methods did not improve model fit and high‐order polynomials are not recommended for many RDD approaches [24], including RDD‐in‐time models [25]. The exposure of interest was an indicator variable identifying individuals who gave birth during or after January 2020. The model included a continuous variable ranging from −24 to 23, which measured when births occurred as the number of months from the January 2020 cutoff. Adjusted analyses controlled for delivery month and state fixed effects, covariates for state Medicaid expansion status, postpartum survey timing, and individual characteristics.

We used triangular kernel weights to more heavily weight observations closer to the cutoff up to 24 months away from January 2020, multiplied by PRAMS survey weights to account for sampling probability, survey design, and nonresponse. This approach allows for all observations to be included, but observations closer to the cutoff received more weight, thereby emphasizing their contribution to the estimated treatment effect. We used robust standard errors clustered at the month‐year level to account for autocorrelation over time, which is recommended for RDDs where time is the running variable [25] (described further in Methods S2).

As the effects of extended Medicaid eligibility on postpartum coverage could vary based on pre‐existing differences in coverage patterns, we conducted stratified RDD analyses to explore potential differences by (1) pre‐FFCRA state Medicaid expansion status, and (2) race and ethnicity for non‐Hispanic Black, Hispanic, and non‐Hispanic White respondents (presented in Figure S1).

### 2.5 Supplemental Analysis

As we were underpowered for stratified analyses among non‐Hispanic Asian or Pacific Islander respondents and non‐Hispanic Indigenous respondents with Medicaid‐paid prenatal care, we included stratified analyses for all racial–ethnic groups among respondents overall as a supplemental analysis (Table S1).

Some respondents may have reported being uninsured despite maintaining Medicaid [18, 26], a phenomenon referred to in the literature as the “Medicaid undercount.” We compared characteristics of respondents who self‐reported postpartum Medicaid versus uninsurance to (1) identify groups who lost coverage despite continuous eligibility who may be at continued risk of coverage loss under state 12‐month postpartum Medicaid extensions, and (2) identify groups who may need additional outreach about postpartum Medicaid (Table S2).

In addition, we conducted several robustness checks for our main models. These include: examining bunching of births around the cutoff to examine manipulation for treatment effects; placebo tests using falsified cutoffs; analyses without triangular kernel weights; alternative mutually exclusive coverage outcomes; alternative standard error clustering by state; donut RDD models omitting births January–March 2020; RDD models using data‐driven narrow bandwidths; and an alternative sample of individuals with Medicaid‐paid deliveries (Figure S2; Tables S3–S9).

Although examining bunching is a standard check for the RDD, bunching in this instance for birth timing is not theoretically possible as individuals did not have advance notice of the PHE or Medicaid policy when conceiving 9 months prior. For the data‐driven narrow bandwidth approach, we employed the “rdrobust” package in Stata to only include observations within a specific narrow bandwidth around the cutoff [27]. We included sensitivity analyses among respondents with Medicaid‐paid deliveries for two reasons. First, the delivery coverage variable is from birth certificate files rather than PRAMS, leading to potential measurement differences; second, individuals with Medicaid‐paid deliveries may differ from those with prenatal Medicaid as some groups receive emergency Medicaid only for delivery, which does not cover the prenatal or postpartum periods [28].

## 3 Results

The study included 46,737 respondents, representing a weighted sample of 2,148,676 postpartum individuals. Table 1 presents the sample characteristics and tests for discontinuities in the characteristics. The largest share of respondents was age 25–29 years (33.7%). The majority of respondents were unmarried in the pre‐period (63.3%), with a significant increase in being married of 3.1 pp at the January 2020 cutoff. In the pre‐period, the majority of respondents resided in urban areas (80.7%), with a significant increase of 2.0 pp at the cutoff. There were no other significant changes in characteristics at the cutoff. Most respondents had educational attainment of high school or less (55.0%), had previous live births (71.3%), completed the survey in English (91.4%), and did not have a preconception health condition (74.4%). Overall, 44.3% of respondents identified as non‐Hispanic White, 26.3% as non‐Hispanic Black, and 19.9% as Hispanic.

**TABLE 1:** Characteristics of respondents with Medicaid‐paid prenatal care before and during the FFCRA Medicaid continuous enrollment provisions, 2018–2021.

	Overal 2018–21, %	Pre‐FFCRA 2018–19, %	During FFCRA 2020–21, %	Discontinuity at 2020, (95% CI)	*p*
Characteristics	*n* = 35,253	*n* = 18,368	*n* = 16,885		
Age at delivery, years					
20–24	30.3	31.4	29.1	−0.8 (−4.0, 2.3)	0.59
25–29	33.7	33.5	33.8	2.1 (−0.4, 4.5)	0.10
30–34	22.7	22.4	23.2	−2.5 (−5.2, 0.2)	0.07
35 or older	13.3	12.7	13.9	1.3 (−1.1, 3.7)	0.29
Marital status					
Married	36.4	36.6	36.2	3.1 (0.7, 5.6)	0.01
Unmarried	63.5	63.3	63.7	−3.1 (−5.6, −0.7)	0.01
Educational attainment					
High school or less	55.0	55.1	54.9	−1.5 (−4.7, 1.7)	0.34
Some college	33.3	34.0	32.6	1.0 (−1.5, 3.4)	0.43
4 years of college or more	10.8	10.1	11.5	0.0 (−0.9, 1.0)	0.93
Urban–rural residence					
Urban	80.4	80.7	80.0	2.0 (0.1, 3.8)	0.04
Rural	19.2	19.3	19.2	−1.6 (−3.4, 0.3)	0.11
Parity					
Primiparous	28.5	28.0	29.0	0.5 (−1.5, 2.4)	0.64
Multiparous	71.3	71.8	70.7	−0.6 (−2.5, 1.4)	0.57
Race and ethnicity					
Asian or Pacific Islander, NH	3.7	3.7	3.7	0.7 (−0.0, 1.5)	0.06
Black, NH	26.3	26.6	25.9	−1.9 (−5.6, 1.9)	0.32
Hispanic	19.9	19.3	20.4	−1.0 (−2.7, 0.8)	0.27
Indigenous, NH	1.0	1.0	1.0	−0.1 (−0.5, 0.3)	0.68
White, NH	44.3	44.5	44.1	2.0 (−1.2, 5.2)	0.22
Other, NH	3.9	3.9	4.0	0.2 (−1.4, 1.8)	0.81
Interview language					
English	91.4	91.6	91.2	−0.1 (−1.5, 1.2)	0.84
Spanish	8.2	7.9	8.4	0.2 (−0.9, 1.4)	0.68
Chinese	0.4	0.5	0.3	−0.1 (−0.6, 0.4)	0.68
Preconception health condition					
Yes	25.4	24.9	25.9	−0.5 (−3.2, 2.1)	0.69
No	74.4	74.9	73.9	0.6 (−2.1, 3.2)	0.68

*Note:* Author's analysis of the Pregnancy Risk Assessment Monitoring System (PRAMS) survey among respondents with Medicaid‐paid prenatal care in 29 jurisdictions, 2018–2021. *n* = 46,737, weighted *N* = 2,148,675.

Abbreviations: CI, confidence interval; FFCRA, Families First Coronavirus Response Act; NH, non‐Hispanic.

**FIGURE 1:**
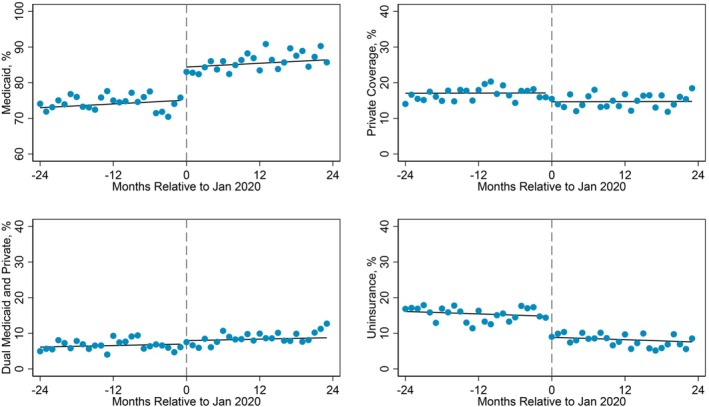
Monthly rates of postpartum insurance coverage before and during the FFCRA Medicaid continuous enrollment provisions, 2018–2021. *Note:* Author's analysis of the Pregnancy Risk Assessment Monitoring System (PRAMS) survey among respondents with Medicaid‐paid prenatal care in 29 jurisdictions, 2018–2021. *n* = 46,737, weighted *N* = 2,148,675. FFCRA is the Families First Coronavirus Response Act. The dashed line indicates the January 2020 cutoff for exposure to extended postpartum Medicaid eligibility.

Figure 1 displays the monthly rates of postpartum insurance types among respondents who gave birth prior to (2018–2019) compared to during extended Medicaid eligibility exposure (2020–2021). During extended Medicaid eligibility, individuals had considerably higher rates of postpartum Medicaid than those prior to the policy. The rates of postpartum uninsurance were also lower after the FFCRA relative to before the policy.

In adjusted RDD models, extended Medicaid eligibility was associated with a 10.7 pp (95% CI: 8.7, 12.6) increase in postpartum Medicaid from a baseline of 74.4%, a 3.5 pp (95% CI: −5.2, −1.8) decrease in private coverage from a baseline of 16.8%, and a 6.5 pp (95% CI: −8.0, −5.0) decrease in uninsurance from a baseline of 15.5% (Table 2). These changes represent a relative 14% increase in Medicaid, a 21% decrease in private coverage, and a 42% decrease in uninsurance. We found no evidence of statistically significant changes in postpartum dual Medicaid and private coverage enrollment associated with extended Medicaid eligibility.

**TABLE 2:** Changes in postpartum insurance coverage during the FFCRA Medicaid continuous enrollment provisions, 2018–21.

Outcome	Baseline coverage rates, 2018–19	Unadjusted discontinuity at 2020 (95% CI)	*p*	Adjusted discontinuity at 2020 (95% CI)	*p*
Medicaid	74.4	9.3 (6.6, 12.0)	< 0.001	10.7 (8.7, 12.6)	< 0.001
Private coverage	16.8	−2.5 (−4.3, −0.7)	0.008	−3.5 (−5.2, −1.8)	< 0.001
Dual medicaid and private	6.6	1.0 (−0.7, 2.6)	0.26	0.7 (−0.7, 2.0)	0.34
Uninsurance	15.5	−5.9 (−7.6, −4.2)	< 0.001	−6.5 (−8.0, −5.0)	< 0.001

*Note:* Author's analysis of the Pregnancy Risk Assessment Monitoring System (PRAMS) survey among respondents with Medicaid‐paid prenatal care in 29 jurisdictions, 2018–2021. *n* = 46,737, weighted *N* = 2,148,675.

Abbreviations: CI, confidence interval; FFCRA, Families First Coronavirus Response Act.

In stratified RDD models, we found larger effect estimates of extended Medicaid eligibility on postpartum Medicaid and uninsurance in non‐expansion states that had lower baseline postpartum Medicaid rates compared to Medicaid expansion states (Table 3). Among non‐expansion states, extended Medicaid eligibility was associated with a 15.5 pp (95% CI: 11.1, 19.9) increase in postpartum Medicaid from a baseline of 59.4%. In Medicaid expansion states, extended Medicaid eligibility was associated with an increase in postpartum Medicaid of 7.9 pp (95% CI: 5.9, 9.8) from a baseline of 81.5%. Postpartum uninsurance decreased by 10.6 pp (95% CI: −14.9, −6.3) in non‐expansion states and by 4.2 pp (95% CI: −5.4, −3.0) in expansion states from baselines of 24.3% and 11.3%, respectively. We did not find differences between expansion and non‐expansion states in the effect of extended eligibility on postpartum private insurance or dual enrollment. However, extended Medicaid eligibility was associated with a 4.3 pp (95% CI: −6.0, −2.5) decrease in postpartum private coverage in expansion states and a 2.5 pp (95% CI: 0.4, 4.5) increase in dual enrollment in non‐expansion states.

**TABLE 3:** Changes in postpartum insurance coverage during the FFCRA Medicaid continuous enrollment provisions by Medicaid expansion status, 2018–21.

Outcome	Baseline coverage rates, 2018–19	Unadjusted discontinuity at 2020 (95% CI)	*p*	Adjusted discontinuity at 2020 (95% CI)	*p*
Medicaid expansion states (*n* = 31,547, weighted *N* = 1,462,021)					
Medicaid	81.5	8.0 (5.8, 10.2)	< 0.001	7.9 (5.9, 9.8)	< 0.001
Private coverage	14.3	−3.2 (−5.1, −1.3)	0.001	−4.3 (−6.0, −2.5)	< 0.001
Dual medicaid and private	7.1	−0.1 (−2.0, 1.8)	0.93	−0.6 (−2.4, 1.1)	0.48
Uninsurance	11.3	−4.9 (−6.3, −3.4)	< 0.001	−4.2 (−5.4, −3.0)	< 0.001
Non‐expansion states (*n* = 15,190, weighted *N* = 686,654)					
Medicaid	59.4	11.6 (7.0, 16.1)	< 0.001	15.5 (11.1, 19.9)	< 0.001
Private coverage	21.9	−0.8 (−4.2, 2.6)	0.63	−2.4 (−5.8, 1.0)	0.16
Dual medicaid and private	5.7	3.1 (0.8, 5.4)	0.01	2.5 (0.4, 4.5)	0.02
Uninsurance	24.3	−7.7 (−11.3, −4.1)	< 0.001	−10.6 (−14.9, −6.3)	< 0.001

*Note:* Author's analysis of the Pregnancy Risk Assessment Monitoring System (PRAMS) survey among respondents with Medicaid‐paid prenatal care in 29 jurisdictions, 2018–2021.

Abbreviations: CI, confidence interval; FFCRA, Families First Coronavirus Response Act.

In models stratified by race and ethnicity, estimates for increases in postpartum Medicaid and decreases in private coverage and uninsurance were significant among each included racial–ethnic group with overlapping confidence levels (Table 4). Extended Medicaid eligibility was associated with increases in postpartum Medicaid of 8.3 pp (95% 5.1, 11.1) among non‐Hispanic Black respondents, 9.7 pp (95% CI: 6.0, 13.4) among Hispanic respondents, and 12.0 pp (95% CI: 9.1, 15.0) among non‐Hispanic White respondents. From baseline levels, these represent a 10% increase among non‐Hispanic Black respondents, a 15% increase among Hispanic respondents, and a 16% increase among non‐Hispanic White respondents. There were significant declines in postpartum uninsurance of 4.4 pp (95% −7.4, −1.4) among non‐Hispanic Black respondents, 5.3 pp (95% CI: −9.5, −1.1) among Hispanic respondents, and 7.7 pp (95% CI: −10.0, −5.4) among non‐Hispanic White respondents, representing declines of 41%, 19%, and 50% from baseline levels, respectively.

**TABLE 4:** Changes in postpartum insurance coverage during the FFCRA Medicaid continuous enrollment provisions by race and ethnicity, 2018–21.

Outcome	Baseline coverage rates, 2018–19	Unadjusted discontinuity at 2020 (95% CI)	*p*	Adjusted discontinuity at 2020 (95% CI)	*p*
Non‐Hispanic Black respondents (*n* = 13,696, weighted *N* = 565,293)					
Medicaid	80.1	7.5 (3.2, 11.9)	0.001	8.3 (5.1, 11.6)	< 0.001
Private coverage	16.0	−3.2 (−6.4, 0.0)	0.05	−4.6 (−8.1, −1.1)	0.01
Dual medicaid and private	6.7	0.0 (−2.1, 2.2)	0.97	−0.7 (−2.6, 1.3)	0.49
Uninsurance	10.7	−4.3 (−7.7, −1.0)	0.01	−4.4 (−7.4, −1.4)	0.005
Hispanic respondents (*n* = 9632, weighted *N* = 426,634)					
Medicaid	64.5	7.6 (1.5, 13.7)	0.02	9.7 (6.0, 13.4)	< 0.001
Private coverage	12.3	−3.7 (−7.8, 0.4)	0.08	−3.4 (−6.3, −0.6)	0.02
Dual medicaid and private	4.8	0.7 (−2.0, 3.4)	0.62	1.0 (−0.9, 2.8)	0.30
Uninsurance	28.0	−3.2 (−9.1, 2.7)	0.28	−5.3 (−9.5, −1.1)	0.02
Non‐Hispanic White respondents (*n* = 14,778, weighted *N* = 952,578)					
Medicaid	75.4	10.5 (7.4, 13.7)	< 0.001	12.0 (9.1, 15.0)	< 0.001
Private coverage	19.2	−2.1 (−5.4, 1.3)	0.22	−3.1 (−6.1, −0.0)	0.05
Dual medicaid and private	7.4	2.0 (−0.3, 4.3)	0.09	1.3 (−0.9, 3.4)	0.24
Uninsurance	12.9	−6.5 (−9.2, −3.8)	< 0.001	−7.7 (−10.0, −5.4)	< 0.001

*Note:* Author's analysis of the Pregnancy Risk Assessment Monitoring System (PRAMS) survey among respondents with Medicaid‐paid prenatal care in 29 jurisdictions, 2018–2021.

Abbreviations: CI, confidence interval; FFCRA, Families First Coronavirus Response Act.

In adjusted stratified models among the sample of PRAMS respondents overall, we found similar effect estimates for coverage changes among non‐Hispanic Black respondents compared to primary models among prenatal Medicaid enrollees (Table S1). Among Hispanic and non‐Hispanic White respondents overall, we found smaller effect estimates for coverage changes than among primary models, and no evidence of changes in postpartum private coverage. We found no evidence of significant changes in postpartum coverage associated with the FFCRA among non‐Hispanic Asian or Pacific Islander respondents overall. Among non‐Hispanic Indigenous respondents overall, we found that extended Medicaid eligibility was associated with an increase in postpartum dual Medicaid and private coverage enrollment by 5.3 pp (95% CI: 1.6, 9.0).

For all primary models—overall, by Medicaid expansion status, and by race and ethnicity—the RDD estimates in adjusted and unadjusted models had overlapping confidence intervals, suggesting that the inclusion of covariates did not substantially alter the estimates. In supplemental analyses, we found significant differences in reporting postpartum Medicaid versus uninsurance by age 35 years or older, marital status, educational attainment, parity, race and ethnicity, interview language, and preconception health condition (Table S2). The largest difference in characteristics between groups was for Hispanic ethnicity; 49.5% of prenatal Medicaid enrollees reporting postpartum uninsurance were Hispanic compared to 18.3% of those reporting postpartum Medicaid. This finding aligns with differences by interview language, as a considerably higher share of respondents who completed the survey in Spanish reported postpartum uninsurance (36.2%) compared to postpartum Medicaid (6.2%).

We found no evidence of manipulation of birth timing around the January 2020 cutoff (Figure S2). Using falsified alternative discontinuity cutoffs, we found no evidence of significant changes in coverage at the two placebo cutoffs (Table S3). Results were similar to main models using PRAMS weights without triangular kernel weights, including alternative postpartum coverage outcomes that were mutually exclusive, and using alternative standard error clustering at the state level (Tables S4–S6).

When omitting individuals whose births occurred January–March 2020 using a donut RDD model, we found results that were similar to main models regarding effect size and statistical significance for postpartum Medicaid, private coverage, and uninsurance (Table S7). However, we found significant increases in dual Medicaid and private coverage in our donut RDD models, which were not observed in main models. In adjusted models employing the alternative data‐driven narrow bandwidth approach, we found results similar to main models for the postpartum Medicaid and dual coverage outcomes (Table S8). However, we found a larger decrease in postpartum uninsurance in these models compared to primary analyses, and no significant decreases in postpartum private coverage. Using the alternative study sample of respondents who had Medicaid‐paid deliveries, we found results that were similar to main models regarding effect sizes and statistical significance (Table S9).

## 4 Discussion

In this RDD analysis of changes in self‐reported postpartum coverage from 2018 to 2021, we found that continuous Medicaid increased by 14% and that uninsurance decreased by 42% among prenatal Medicaid enrollees during the continuous enrollment provisions. As these estimates relied on self‐report, it is possible that increases in Medicaid and declines in uninsurance may have been even larger than estimated results if PRAMS data were subject to the “Medicaid undercount” seen in other survey data [18, 26].

Our results are consistent with previous research finding postpartum Medicaid increases during the continuous enrollment provisions [13, 14, 15, 16, 17]. While most previous literature used pre‐post study designs [13, 14, 15], we used an RDD, a rigorous quasi‐experimental study design exploiting variation in policy exposure over time to provide a national estimate of the policy's impact. Our study is most similar to a study that used a continuous DID design to examine the impact of continuous enrollment on postpartum insurance and healthcare receipt, which found that a 100% FPL change in eligibility increased postpartum Medicaid enrollment [16]. However, while the previous study found no effect on postpartum uninsurance or private insurance, we found that extended Medicaid eligibility was associated with a decline in transitioning to uninsurance and private insurance [16]. The degree to which increased Medicaid represents a substitution away from private insurance, and the relative benefits of public compared to private postpartum insurance, is an important area for future work. In addition, we found significant increases in postpartum Medicaid among non‐Hispanic Black, Hispanic, and non‐Hispanic White respondents, while this previous research found Medicaid increases only among non‐Hispanic White respondents. Finally, we found significant differences by state Medicaid expansion status, which was not examined in this previous paper.

Our results suggest that residents of non‐expansion states, where postpartum Medicaid loss was higher prior to the FFCRA, experienced larger declines in postpartum uninsurance than residents of Medicaid expansion states. These findings indicate that individuals in the remaining 10 non‐expansion states [29], 9 of which have implemented 12‐month postpartum Medicaid extensions under ARPA [12], may benefit more from extended postpartum Medicaid relative to individuals in states with Medicaid expansion. The remaining non‐expansion state to not have implemented a 12‐month postpartum Medicaid extension, Wisconsin, is extending Medicaid up to only 90 days postpartum rather than through the postpartum year, although a 12‐month extension is under consideration [12].

Among included racial–ethnic groups in primary models, we found similar estimates for increases in postpartum Medicaid and decreases in uninsurance. However, these estimates reflect a decrease in uninsurance of 41% among non‐Hispanic Black respondents and 50% among non‐Hispanic White respondents relative to a 19% reduction among Hispanic respondents, who had the highest baseline levels of uninsurance. As a result, our findings indicate that inequities in postpartum uninsurance will likely persist between Hispanic birthing people and non‐Hispanic birthing people despite extended postpartum Medicaid.

Hispanic respondents and respondents who completed the survey in Spanish comprised a disproportionate share of respondents who continued to report uninsurance while continuous enrollment provisions were in place. Our results align with prior research using 2016–20 PRAMS data, which found that Spanish‐speaking Hispanic respondents reported lower perinatal care use than English‐speaking Hispanic respondents, potentially driven by higher rates of perinatal uninsurance [30]. Individuals who received public coverage through certain pathways such as emergency Medicaid or the CHIP unborn child option typically were not eligible for continuous enrollment under the FFCRA based on immigration status [19]. Therefore, these findings may reflect higher rates of transitioning from prenatal Medicaid to postpartum uninsurance among Hispanic prenatal Medicaid enrollees due to ineligibility based on immigration status. Alternatively, this group may have had more limited information about continuous enrollment, suggesting that culturally tailored information available in multiple languages is needed to increase awareness of extended Medicaid eligibility.

Our findings likely reflect the current policy environment since nearly all states have adopted 12‐month postpartum Medicaid extensions. However, as extended Medicaid eligibility was insufficient to eliminate postpartum uninsurance entirely and high rates of uninsurance persisted among Hispanic respondents, states should consider additional policies. These policies could include increasing the income eligibility thresholds for parental or pregnancy‐related Medicaid and expanding Medicaid or Marketplace coverage to non‐qualifying immigrant groups.

It is especially urgent to improve insurance stability and care access after childbirth given the inequitable and increasing rates of maternal morbidity and mortality in the US [1, 2]. Over half of pregnancy‐related deaths occur between 1 day and 1 year postpartum, making access to insurance crucial during this time [31]. Improvements in coverage continuity could have meaningful implications for access to postpartum care. Previous research suggests that discontinuous postpartum insurance can decrease postpartum healthcare use [32, 33]. However, postpartum insurance may not be sufficient to ensure access to high‐quality care, particularly for people of color and residents of rural areas [34, 35, 36]. Future analyses could examine outcomes beyond those captured in PRAMS, which only includes postpartum visits that occurred at a period already covered by Medicaid before the policy change. Increased postpartum coverage may not have translated into increased care access, particularly during the PHE due to pandemic‐related disruptions in healthcare, including during the perinatal period [37, 38, 39]. Although the FFCRA offers early evidence of potential coverage impacts under state 12‐month postpartum Medicaid extensions, it will be important to assess how these postpartum extensions impact care access and maternal health outcomes outside of the PHE context.

As of April 2023, states began disenrolling Medicaid recipients again under the “unwinding” of the continuous enrollment provisions [40]. It will be critical to monitor how the end of these provisions affected postpartum Medicaid continuity and whether it exacerbated existing inequities in Medicaid loss [41]. As the state 12‐month postpartum Medicaid extensions were likely protective against postpartum Medicaid loss, this monitoring will be particularly important in the two states that have not yet adopted 12‐month extensions: Wisconsin and Arkansas [12]. In Arkansas, the only state that has not adopted even a limited coverage extension, the unwinding could have resulted in a return to pre‐FFCRA levels of coverage inequities and postpartum Medicaid loss. Analyses of Arkansas data indicate that after Medicaid expansion, 20% of individuals with Medicaid‐financed births lacked continuous Medicaid enrollment through 6 months postpartum, highlighting the necessity for additional efforts to promote postpartum Medicaid continuity in the state [7].

Our study had several limitations. Our study design examined changes associated with extended Medicaid eligibility that occurred concurrently with the pandemic, making it hard to disentangle the effects of the Medicaid policy from those of the pandemic itself. While there was pandemic‐related job loss that could affect employer‐sponsored private insurance, the major mechanism for changes in coverage stability for prenatal Medicaid recipients during the PHE was the continuous enrollment provision. Changes in employer‐sponsored insurance did not occur immediately with the start of the PHE, with research showing largely stable employer‐sponsored coverage in the first year of the pandemic despite pandemic‐related labor market disruptions [42]. Additional limitations include that our study's use of self‐reported insurance may have resulted in an underestimate of the FFCRA's impact. However, use of self‐reported outcomes also allowed us to explore the “Medicaid undercount.” In addition, PRAMS data is collected typically 2–6 months after childbirth, which only reflects coverage in the early postpartum period. The 29 jurisdictions included in our study may not be generalizable nationally. Finally, we had limited statistical power to examine differences by race and ethnicity, and we did not have sufficient sample size to consider coverage changes among prenatal Medicaid enrollees who were Asian or Pacific Islander, Indigenous, or other racial–ethnic groups, which is an important area for future research.

In conclusion, this study found significant gains in postpartum Medicaid continuity during the continuous enrollment provisions. These findings suggest that postpartum Medicaid stability will increase under the ARPA 12‐month state postpartum Medicaid extensions that have been adopted by 48 states and DC. However, our findings indicate persistent racial–ethnic postpartum coverage disparities for Hispanic birthing people, potentially indicating that additional efforts are needed beyond coverage extensions to address coverage inequities and ultimately racial–ethnic disparities in maternal health.

## Conflicts of Interest

The authors declare no conflicts of interest.

## Supporting information


Data S1.
