# Supplementary material for: Association of COVID‐19 Continuous Enrollment With Self‐Reported Postpartum Medicaid Continuity and Coverage Inequities
Source: Health Serv Res. 2025 Mar 26;60(4):e14618. doi: 10.1111/1475-6773.14618 (PMC12277109; doi:10.1111/1475-6773.14618)
Supplement: Supplementary file 1 — Data S1. [file HESR-60-0-s001.pdf]

Supplemental Material

Supplemental Methods 1. Study Specifications: PRAMS State Sample
Supplemental Methods 2. Study Specifications: Regression Discontinuity Design Model
Supplemental Figure 1. Stratified Plots of Monthly Postpartum Coverage Rates
Supplemental Table 1. Stratified Analyses by Race and Ethnicity Among Overall Population
Supplemental Table 2. Characteristics of Respondents with Prenatal Medicaid Reporting Postpartum Medicaid and Uninsurance During the PHE
Supplemental Figure 2. Specification Check: Bunching at the Cutoff
Supplemental Table 3. Sensitivity Analysis: Placebo Discontinuity Cutoff
Supplemental Table 4. Sensitivity Analysis: Model without Triangular Kernel Weights
Supplemental Table 5. Sensitivity Analysis: Alternative Postpartum Coverage Outcomes
Supplemental Table 6. Sensitivity Analysis: Alternative Standard Error Clustering
Supplemental Table 7. Sensitivity Analysis: Donut Regression Discontinuity
Supplemental Table 8. Sensitivity Analysis: Data-Driven Narrow Bandwidth Approach
Supplemental Table 9. Sensitivity Analysis: Population of Medicaid-Paid Deliveries

Supplemental Methods 1. Study Specifications: PRAMS State Sample

To ensure that an inconsistent state composition of the sample was not driving any change in outcomes over time, we restricted the study to states that consistently met survey response rate criteria and thus had available data throughout the study period. This approach resulted in a study sample comprised of 29 jurisdictions, with an annual median weighted response rate ranging from 56.4% to 60.5%.

	Weighted PRAMS Response Rate			
Jurisdiction	2021	2020	2019	2018
Alabama	49.7	55.2	56.4	54.2
Arkansas	52.9	58.3	55.6	52.0
Colorado	60.2	63.3	59.4	61.6
Connecticut	58.9	63.2	55.7	58.4
District of Columbia	54.6	58.6	51.9	51.4
Delaware	52.1	53.7	57.4	59.4
Georgia	50.1	53.1	61	59.2
Illinois	56.4	61.1	59.3	61.4
Kansas	63.4	65.8	63.3	60.8
Louisiana	56.8	57.3	57.1	64.3
Massachusetts	57	59.6	61.2	62.4
Michigan	57	59.2	55.6	57.7
Minnesota	52.1	54.2	54.7	56.8
Mississippi	54.4	60.4	65	60.2
Missouri	50.1	57.1	56.9	56.8
Montana	55.1	53.1	51.3	53
Nebraska	59.4	66.0	63.8	60.6
New Jersey	56.3	64.8	65.3	67.3
New Mexico	62.2	61.7	66.6	59.8
New York City	59.2	61.3	60.8	64.7
North Dakota	54.1	60.5	59.1	59.9
Oregon	56.8	64.2	69.3	52.5
Pennsylvania	56.1	60.3	58.2	61.2
South Dakota	65.9	66.8	68.1	64.3
Utah	58.3	66.9	72.8	62.2
Vermont	62.9	66.1	62.4	68.8
Washington	60.6	64.4	64.1	61.5
Wisconsin	55.8	64.1	59.7	55
Wyoming	54.2	51.4	55	61.8
Median	56.4	60.5	59.4	60.2

Supplemental Methods 2. Study Specifications: Regression Discontinuity Design Model

Model Specifications

We estimated the following model for our primary regression discontinuity design:

$$\Upsilon_{iST} = \beta_1 Treated_T + \beta_2 CutoffDistance_T + \delta Month_T + \delta State_S + \chi_{iST} + \in i_{ST}$$

The outcome of interest is Υ_{ST}. The main treatment effect is $Treated_T$, representing whether or not the 60 day postpartum period occurred during the COVID-19 continuous coverage provisions (births starting January 2020). $\beta_2 CutoffDistance_T$ is the distance in months from January 2020, the cutoff for the primary regression discontinuity design. The model includes delivery month fixed effects ($\delta Month_T$), state fixed effects ($\delta State_S$), and a vector of covariates for state Medicaid expansion status, age, marital status, education, parity, rurality, postpartum survey timing, preconception health conditions, survey language, and race and ethnicity (χ_{ST}). We used robust standard errors clustered by time at the month-year level, which is recommended for regression discontinuities where time is the running variable.

To select the appropriate polynomial order for the global polynomial model, we added quadratic and cubic terms to the above linear regression model. We used an F test to determine whether these higher-order terms significantly improved the model fit. This approach give preference to the simplest functional form possible, unless these is clear evidence that the added complexity of the quadratic or cubic terms is warranted. The F test results indicated that the addition of quadratic and cubic terms did not significantly improve the model fit. As a result, we used the polynomial linear regression models for our main models for the regression discontinuity design.

We used triangular kernel weights to more heavily weight observations closer to the January 2020 cutoff up to 24 months away from the cutoff, and PRAMS survey weights to account for sampling probability, survey design, and nonresponse in the survey. This approach ensures that all observations are included for the global parametric model, but observations closer to the cutoff receive more weight, thereby emphasizing their contribution to the estimated treatment effect. The triangular kernel weights were calculated as follows:

$$\text{Kernel\_weight}_i = \max\ (0{,}1 - \frac{CutoffDistance_i}{24})$$

These triangular kernel weights were then multiplied by the PRAMS survey weights (WTANAL) to produce the combined weights used in the regression:

$$\text{Combined\_weight}_i = WTANAL_i * Kernel\_weight_i$$

Supplemental Figure 1. Stratified Plots of Monthly Postpartum Coverage Rates
Medicaid Expansion States

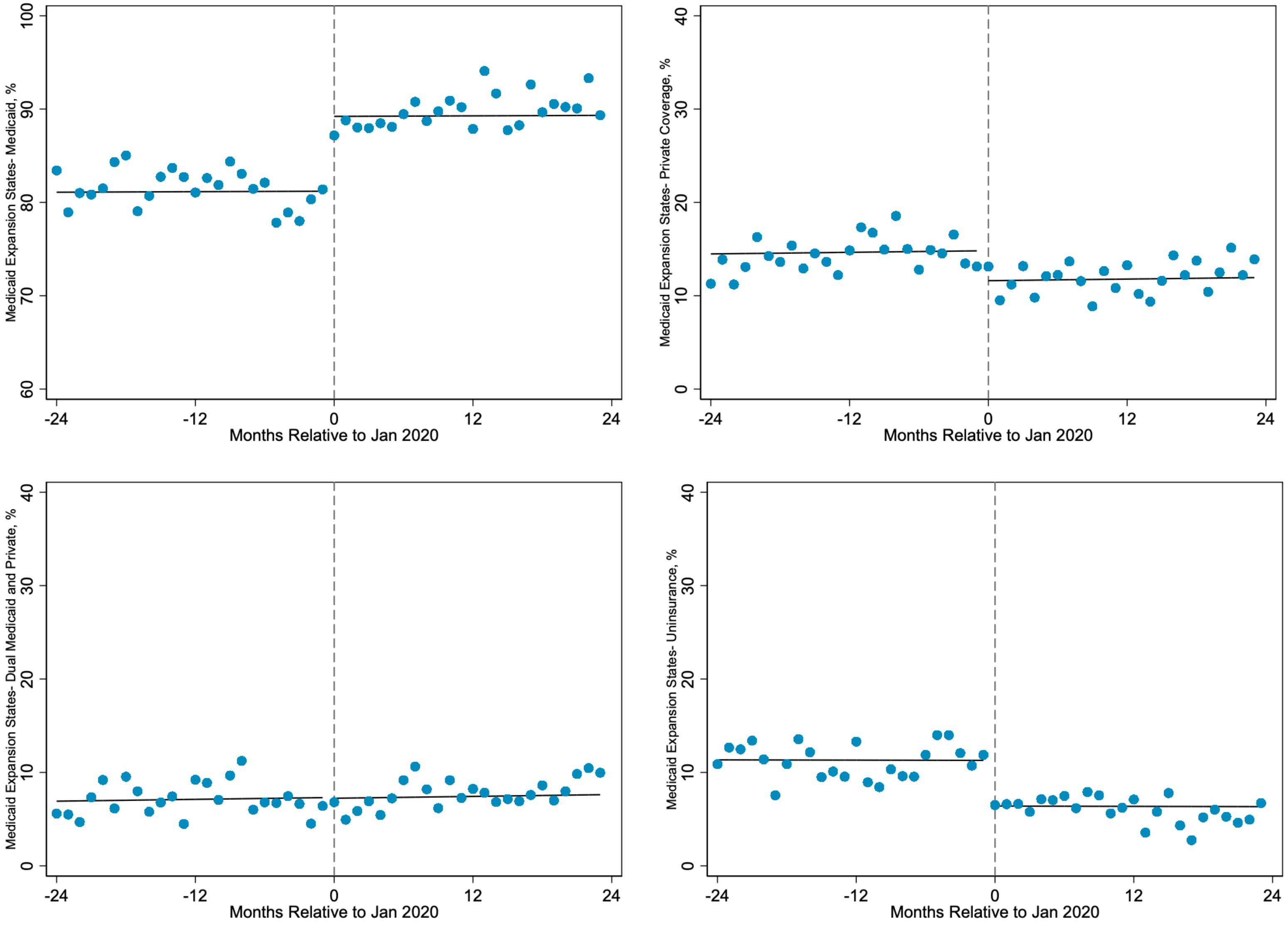

Non-Expansion States

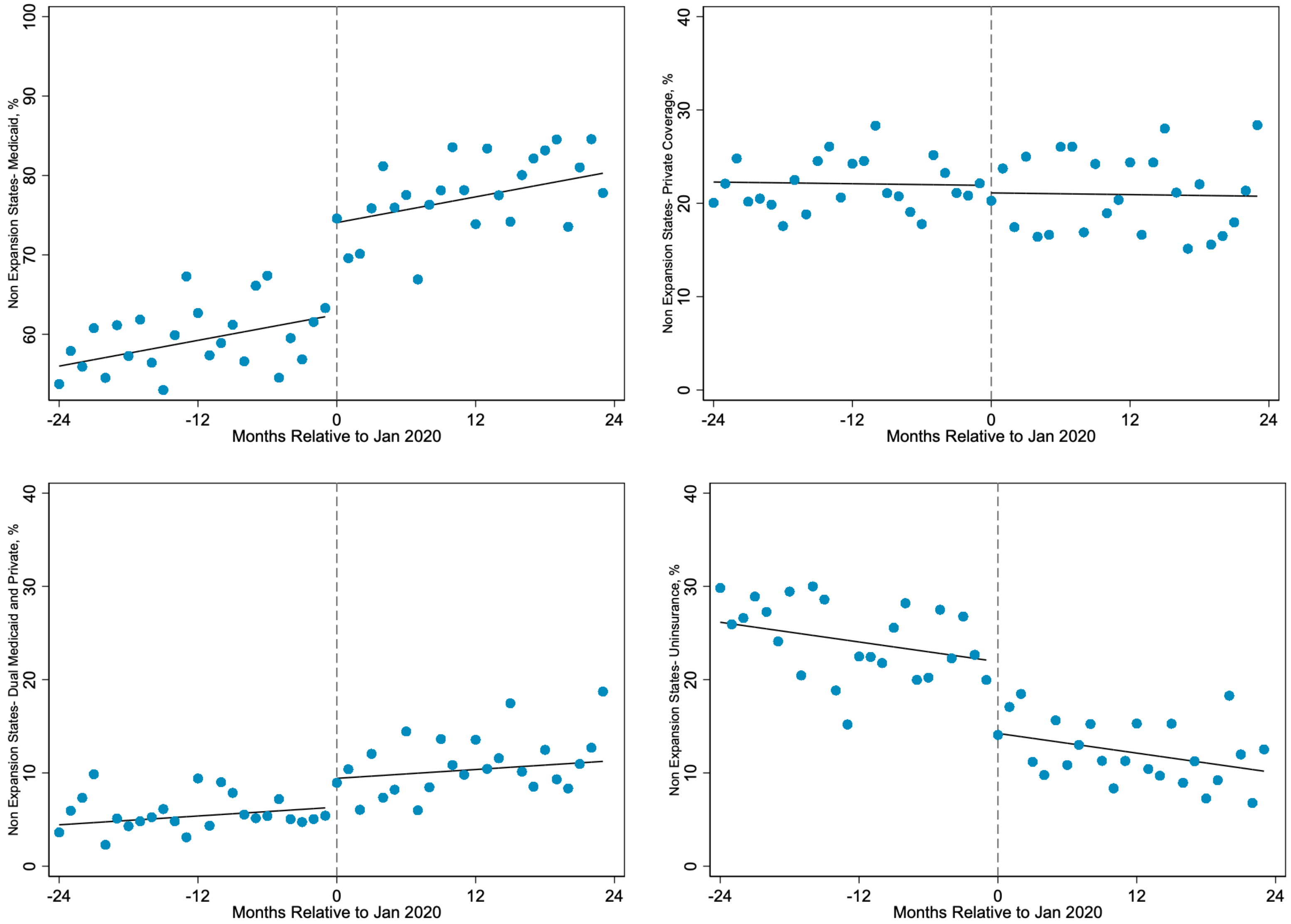

Non-Hispanic Black Respondents

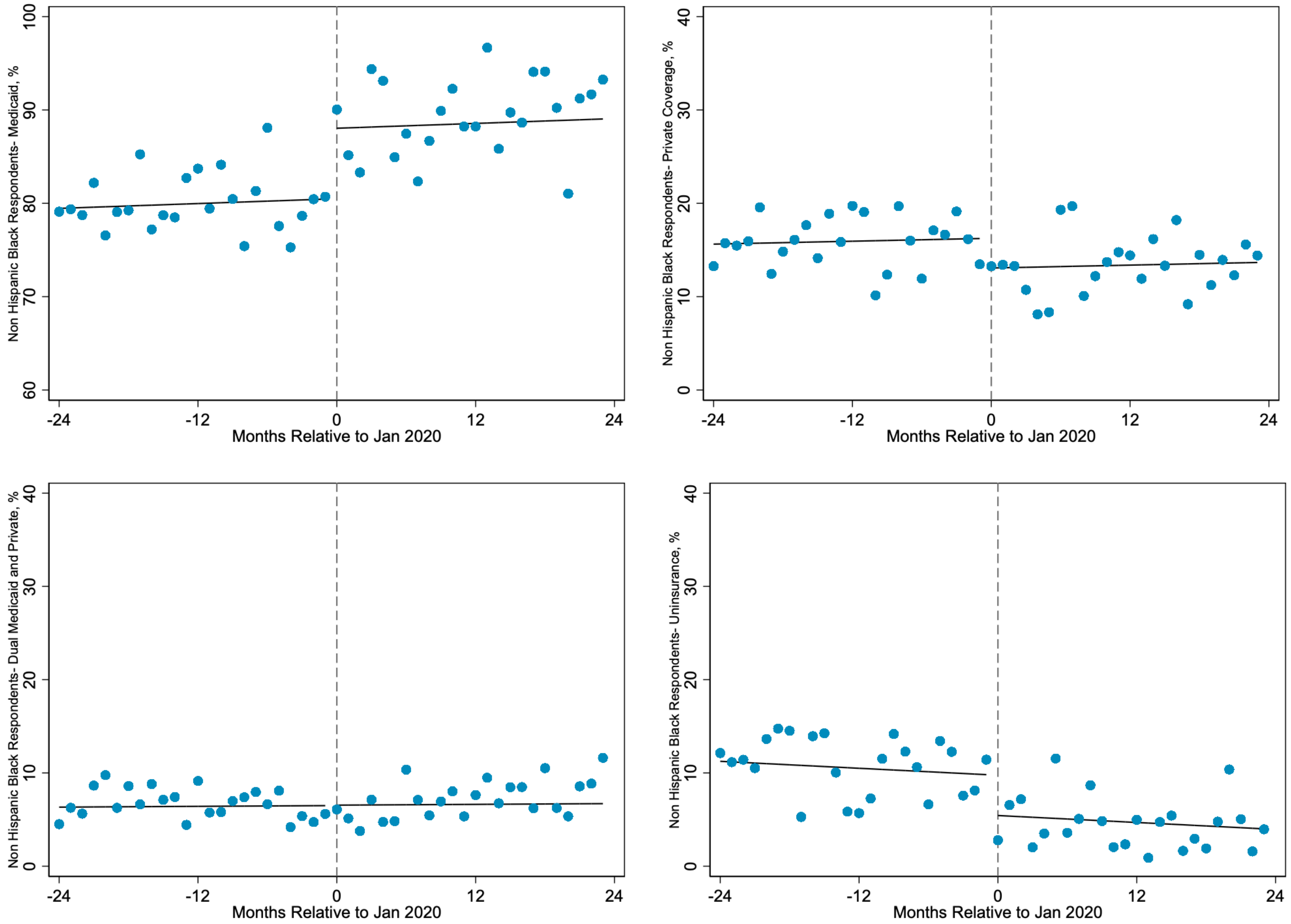

Hispanic Respondents

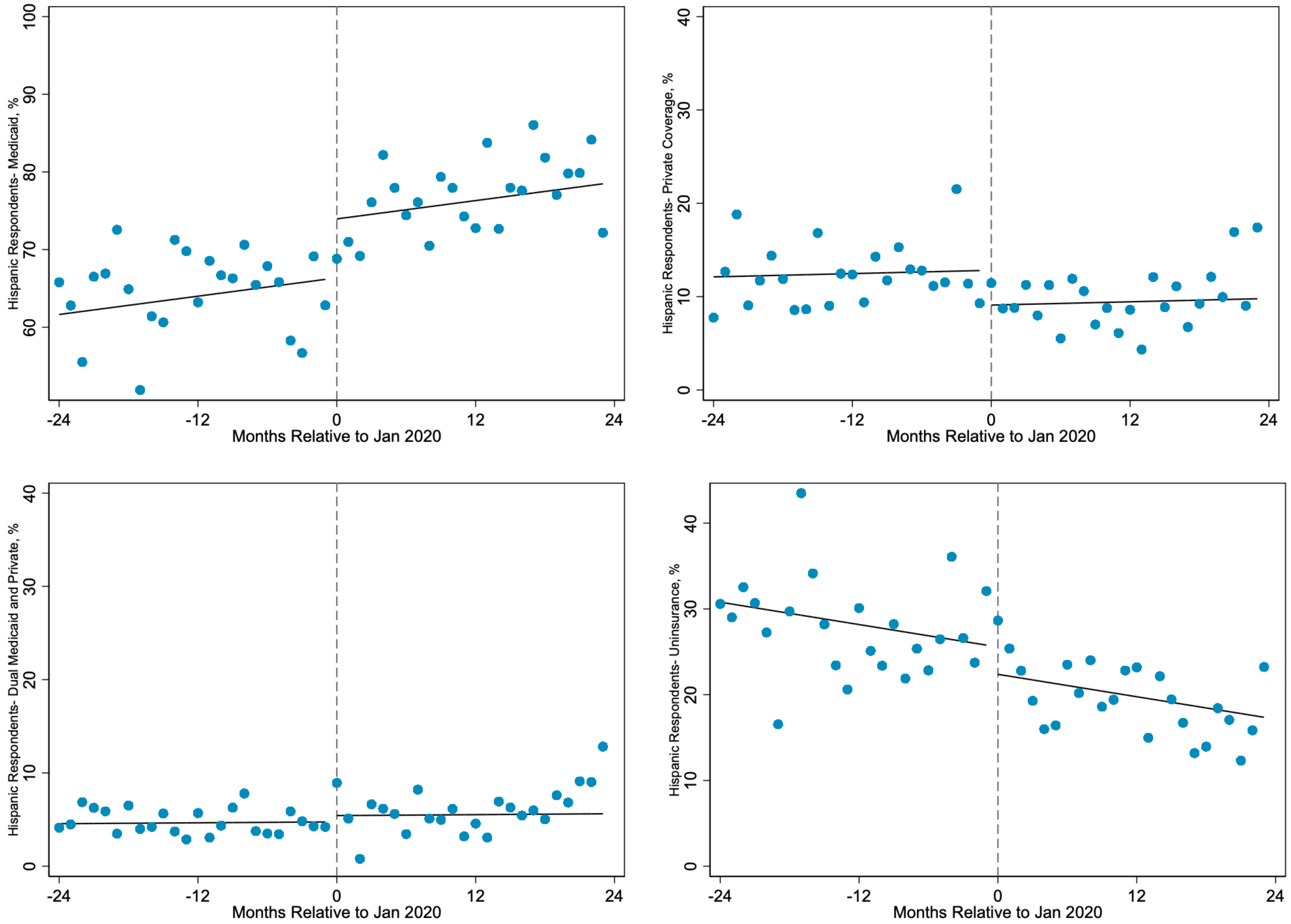

Non-Hispanic White Respondents

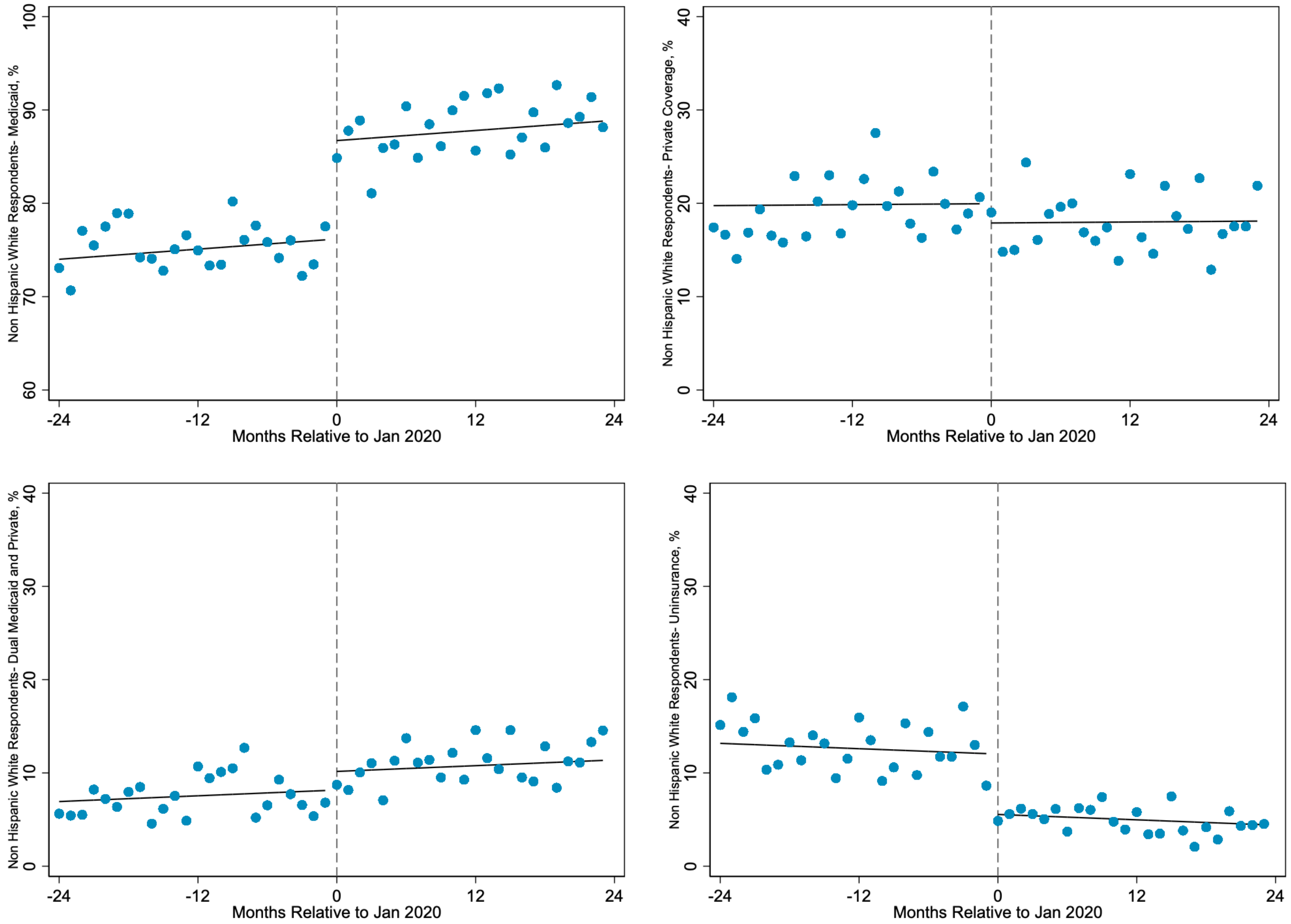

Supplemental Table 1. Stratified Analyses by Race and Ethnicity Among Overall Population

In primary analyses among the sample of respondents with Medicaid-paid prenatal care, we were underpowered to include stratified models among respondents who were non-Hispanic Asian or Pacific Islander or Indigenous. In adjusted stratified models among the sample of PRAMS respondents overall, we found similar effect estimates for coverage changes among non-Hispanic Black respondents compared to primary models among prenatal Medicaid enrollees. Among Hispanic and non-Hispanic White respondents overall, we found smaller effect estimates for coverage changes than among primary models among prenatal Medicaid enrollees, and no evidence of changes in postpartum private coverage. Among non-Hispanic Asian or Pacific Islander respondents overall, we found no evidence of significant changes in postpartum coverage associated with the FFCRA at $p<0.05$. Among non-Hispanic Indigenous respondents overall, we found that extended Medicaid eligibility during the FFCRA was associated with an increase in postpartum dual Medicaid and private coverage enrollment.

Outcome	Baseline Coverage Rates, 2018-19	Unadjusted Discontinuity at 2020 (95% CI)	*P* value	Adjusted Discontinuity at 2020 (95% CI)	*P* value
Non-Hispanic Asian or Pacific Islander Respondents					
Medicaid	22.97	3.63 (-1.05, 8.31)	0.13	2.49 (-1.92, 6.90)	0.26
Private Coverage	73.26	-1.01 (-6.28, 4.26)	0.70	0.77 (-3.33, 4.86)	0.71
Dual Medicaid and Private	1.93	-0.30 (-2.47, 1.88)	0.79	0.71 (-1.42, 2.84)	0.51
Uninsurance	5.71	-2.92 (-5.86, 0.03)	0.05	-2.55 (-5.12, 0.02)	0.05
Non-Hispanic Black Respondents					
Medicaid	60.73	6.87 (2.29, 11.45)	0.004	9.00 (6.41, 11.59)	<.001
Private Coverage	35.05	-3.51 (-8.28, 1.26)	0.15	-5.54 (-8.46, -2.62)	<.001
Dual Medicaid and Private	5.47	-0.08 (-1.90, 1.75)	0.93	-0.73 (-2.56, 1.10)	0.42
Uninsurance	9.69	-3.44 (-5.09, -1.80)	<.001	-4.19 (-6.25, -2.13)	<.001
Hispanic Respondents					
Medicaid	38.61	2.25 (-1.38, 5.88)	0.22	4.42 (1.89, 6.95)	0.001
Private Coverage	35.42	0.62 (-3.56, 4.81)	0.77	1.18 (-1.11, 3.46)	0.30
Dual Medicaid and Private	3.14	0.69 (-0.90, 2.29)	0.39	0.60 (-0.64, 1.83)	0.34
Uninsurance	29.12	-2.18 (-5.17, 0.81)	0.15	-5.00 (-7.70, -2.31)	0.001

Non-Hispanic Indigenous Respondents					
Medicaid	66.32	1.79<br>(-11.28, 14.85)	0.78	1.72<br>(-5.80, 9.24)	0.65
Private Coverage	22.65	-1.76<br>(-14.60, 11.09)	0.78	0.67<br>(-5.86, 7.19)	0.84
Dual Medicaid and Private	3.00	2.89<br>(-0.90, 6.68)	0.13	5.27<br>(1.56, 8.99)	0.006
Uninsurance	14.04	2.86<br>(-4.89, 10.60)	0.46	2.89<br>(-2.64, 8.41)	0.30
Non-Hispanic White Respondents					
Medicaid	24.84	4.22<br>(2.71, 5.73)	<.001	4.83<br>(3.52, 6.15)	<.001
Private Coverage	71.50	-0.87<br>(-2.69, 0.95)	0.34	-0.94<br>(-2.30, 0.42)	0.17
Dual Medicaid and Private	2.74	0.74<br>(-0.03, 1.51)	0.06	0.74<br>(0.01, 1.47)	0.05
Uninsurance	6.40	-2.62<br>(-4.00, -1.23)	<.001	-3.15<br>(-4.19, -2.11)	<.001

Supplemental Table 2. Characteristics of Respondents with Prenatal Medicaid Reporting Postpartum Medicaid and Uninsurance During the PHE

This table presents characteristics of respondents who self-reported postpartum Medicaid and respondents who self-reported postpartum uninsurance among a sample of individuals who self-reported prenatal Medicaid coverage in order to understand difference in characteristics of awareness of postpartum Medicaid coverage. Research has found that the Medicaid `undercount,' or reporting uninsurance despite being enrolled in Medicaid, was prevalent during the COVID-19 continuous enrollment provisions.[1] However, some prenatal Medicaid recipients who reported postpartum uninsurance likely did lose postpartum coverage due to reasons such as immigration status or moving states. By focusing on a sample of self-reported prenatal Medicaid recipients from PRAMS rather than Medicaid-paid deliveries from the birth certificate files, we have tried to minimize factors such as emergency Medicaid receipt for labor and delivery or coverage differences due to variations in the data source. Understanding characteristics of respondents who self-reported postpartum Medicaid compared to uninsurance can provide insight for 1) identifying groups who did lose coverage despite continuous eligibility policies that were in place, who will likely continue to lose postpartum Medicaid under state postpartum Medicaid extensions, and 2) targeting groups who may need additional information and communication to increase awareness about their coverage enrollment so that the benefits of continuous coverage can be realized through improved health care access.

We found significant differences in reporting postpartum Medicaid by age 35 years or older, marital status, educational attainment, parity, race and ethnicity, interview language, and preconception health condition. Despite prior research indicating significant rural-urban differences in perinatal insurance, we found no evidence of significant differences in reporting postpartum Medicaid by rurality.[2] Hispanic respondents comprised a substantially larger share of prenatal Medicaid enrollees reporting postpartum uninsurance compared to those reporting postpartum Medicaid coverage. This finding aligns with differences by interview language, where a higher share of respondents who completed the survey in Spanish reported postpartum uninsurance compared to postpartum Medicaid. These differences may be driven both by a need for culturally tailored information available in multiple languages, as well as potential true enrollment differences due to ineligibility based on immigration status. Differences by preconception health condition, with comparatively higher reports of postpartum Medicaid among individuals with a preconception health condition, could suggest greater awareness of enrollment among individuals with health conditions. This may be due to increased engagement with the health care system, which could lead to better awareness and understanding of their coverage due to healthcare provider assistance or a higher reliance on their coverage for care.

Characteristics	Self-Reported Postpartum Medicaid During PHE 2020-21, %	Self-Reported Postpartum Uninsurance During PHE 2020-21, %	Difference (95% CI)	*P*-value
Age at Delivery, Years				
20-24	29.21	27.37	1.84 (-0.37, 4.05)	0.10
25-29	33.77	33.16	0.61	0.60

			(-1.69, 2.91)	
30-34	23.14	23.39	-0.25 (-2.30, 1.80)	0.81
35 or older	13.88	16.09	-2.20 (-3.89, -0.51)	0.01
Marital Status				
Married	34.32	41.81	-7.49 (-9.81, -5.18)	<.001
Unmarried	65.59	57.86	7.72 (5.41, 10.04)	<.001
Educational Attainment				
High School or Less	55.02	65.61	-10.59 (-13.00, -8.18)	<.001
Some College	33.20	24.18	9.02 (6.75, 11.29)	<.001
4 Years of College or More	10.89	9.09	1.80 (0.30, 3.30)	0.02
Urban-Rural Residence				
Urban	80.41	79.13	1.28 (-0.65, 3.21)	0.20
Rural	18.83	19.91	-1.08 (-2.98, 0.82)	0.27
Parity				
Primiparous	29.17	25.83	3.35 (1.15, 5.55)	0.003
Multiparous	70.58	74.13	-3.55 (-5.75, -1.34)	0.002
Race and Ethnicity				
Asian or Pacific Islander, NH	3.68	2.49	1.20 (0.30, 2.10)	0.009
Black, NH	26.86	15.37	11.50 (9.38, 13.62)	<.001
Hispanic	18.28	49.54	-31.26 (-33.20, -29.33)	<.001
Indigenous, NH	1.01	0.93	0.08 (-0.40, 0.56)	0.74
White, NH	45.28	26.84	18.44 (16.04, 20.83)	<.001
Other, NH	4.04	3.88	0.16 (-0.79, 1.12)	0.74
Interview Language				
English	93.47	63.68	29.79 (28.45, 31.12)	<.001
Spanish	6.16	36.17	-30.02 (-31.32, -28.71)	<.001

Chinese	0.37	0.14	0.23<br>(-0.06, 0.51)	0.12
Preconception Health Condition				
Yes	27.39	16.66	10.74<br>(8.60, 12.87)	<.001
No	72.44	83.34	-10.90<br>(-13.04, -8.76)	<.001

Supplemental Figure 2. Specification Check: Bunching at the Cutoff

This figure presents the number of births by month among our sample of prenatal Medicaid enrollees age 20 and older for the period 2018-2021. By examining the distribution of births, we can confirm that observations are not clustering around the January 2020 cutoff due to individuals manipulating their treatment exposure. We see no evidence of respondents manipulating the month of their birth for treatment exposure, supporting the assumption for our model that any differences in outcomes observed at January 2020 are likely due to the effects from the Families First Coronavirus Response Act Medicaid continuous enrollment provisions rather than manipulation among the study population.

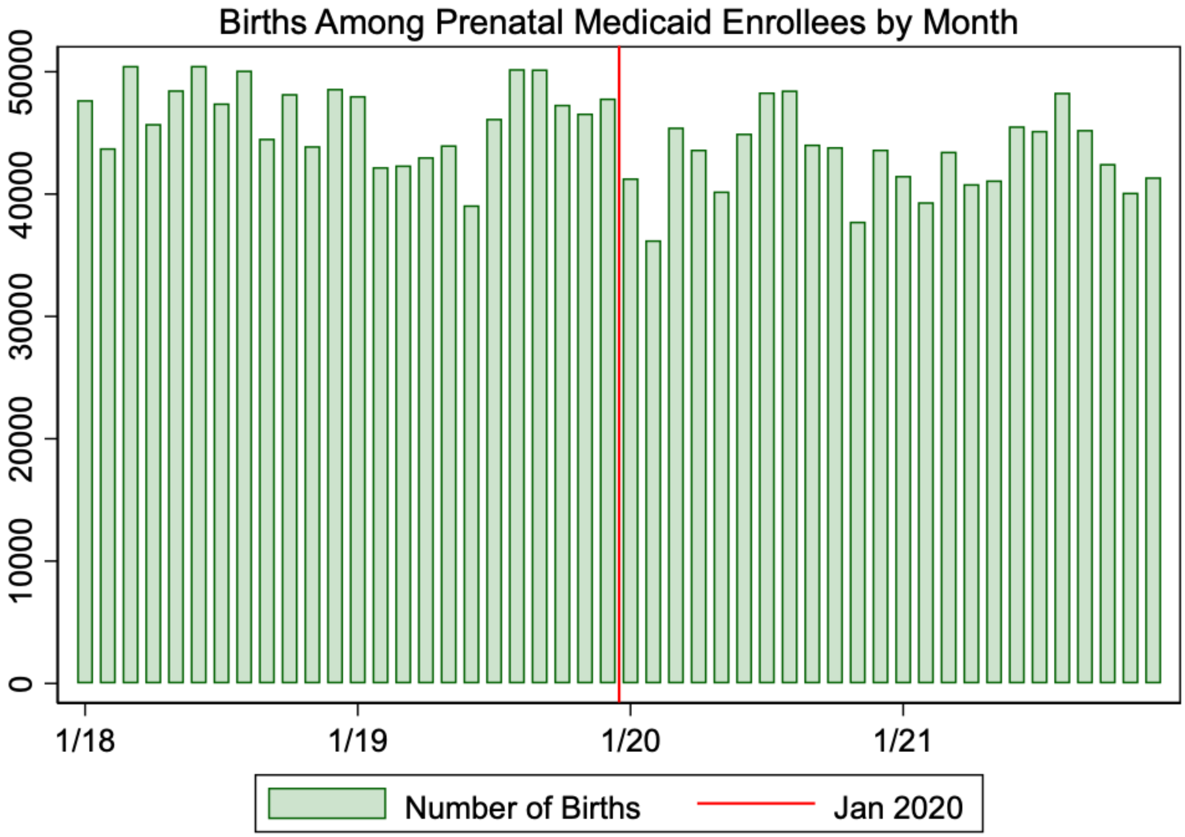

Supplemental Table 3. Sensitivity Analysis: Placebo Discontinuity Cutoff

This sensitivity analysis presents regression discontinuity models using two alternative placebo discontinuity cutoffs for the regression discontinuity design, one below and one above the primary cutoff of January 2020. This approach helps confirm that any significant findings in the primary specifications are not the result of random variation in the data but are actually attributable to changes in postpartum coverage associated with the Families First Coronavirus Response Act. We found no evidence of statistically significant changes in postpartum coverage outcomes at the two placebo discontinuity cutoffs, July 2018 and November 2020.

Outcome	Adjusted Discontinuity at 7/18 (95% CI)	*P* value	Adjusted Discontinuity at 11/20 (95% CI)	*P* value
Medicaid	-1.67 (-3.93, 0.60)	0.15	-2.62 (-5.95, 0.71)	0.12
Private Coverage	0.59 (-1.42, 2.60)	0.56	0.35 (-1.48, 2.18)	0.70
Dual Medicaid and Private	-0.87 (-2.22, 0.49)	0.20	-0.59 (-1.89, 0.72)	0.37
Uninsurance	0.21 (-1.64, 2.05)	0.82	1.68 (-0.23, 3.60)	0.08

Supplemental Table 4. Sensitivity Analysis: Model without Triangular Kernel Weights

This sensitivity analysis presents regression discontinuity models without triangular kernel weights, using only PRAMS survey weights. This approach can confirm that weighting respondents more around the January 2020 cutoff is not the sole driver of our findings. We found results that were consistent with main models with respect to effect sizes and statistical significance.

Outcome	Unadjusted Discontinuity at 2020 (95% CI)	*P* value	Adjusted Discontinuity at 2020 (95% CI)	*P* value
Medicaid	9.20 (6.52, 11.89)	<.001	10.56 (8.63, 12.48)	<.001
Private Coverage	-3.35 (-5.26, -1.43)	0.001	-3.99 (-5.93, -2.04)	<.001
Dual Medicaid and Private	0.43 (-1.34, 2.20)	0.63	0.48 (-1.06, 2.02)	0.54
Uninsurance	-5.43 (-7.17, -3.68)	<.001	-6.10 (-7.63, -4.57)	<.001

Supplemental Table 5. Sensitivity Analysis: Alternative Postpartum Coverage Outcomes

For our primary specifications, postpartum health insurance was categorized as Medicaid, private or military coverage, dual Medicaid and private or military coverage, and uninsurance or only Indian Health Service coverage. In these outcome categories, respondents were allowed to report more than one postpartum coverage type. As a sensitivity analysis, we created mutually exclusive postpartum insurance categories for individuals with Medicaid and individuals with private coverage that excluded individuals who reported dual enrollment. This recategorization did not change estimates for dual enrollment in Medicaid and private or military coverage or for uninsurance or Indian Health Service coverage only. In these analyses, we found estimates that were similar to main models for effect size and statistical significance.

Outcome	Baseline Coverage Rates, 2018-19	Unadjusted Discontinuity at 2020 (95% CI)	*P* value	Adjusted Discontinuity at 2020 (95% CI)	*P* value
Medicaid Only	67.75	8.35 (5.81, 10.90)	<.001	10.00 (8.00, 12.01)	<.001
Private Coverage Only	10.13	-3.43 (-4.95, -1.92)	<.001	-4.17 (-5.66, -2.67)	<.001
Dual Medicaid and Private	6.65	0.95 (-0.74, 2.64)	0.26	0.66 (-0.72, 2.04)	0.34
Uninsurance	15.47	-5.87 (-7.60, -4.15)	<.001	-6.50 (-7.97, -5.04)	<.001

Supplemental Table 6. Sensitivity Analysis: Alternative Standard Error Clustering

In our main models, we clustered standard errors by time at the month-year level, which is recommended in regression discontinuity models where time is the running variable.[3] We conducted a sensitivity analysis using an alternative approach of clustering standard errors by state instead, which accounts for potential within-state correlation over time. Using an alternative approach of clustering standard errors helps to ensure that our results are not sensitive to our primary model specifications. Using this approach, we found no meaningful change in statistical significance in adjusted models, suggesting our results are robust to different standard error clustering methods.

Outcome	Unadjusted Discontinuity at 2020 (95% CI)	*P* value	Adjusted Discontinuity at 2020 (95% CI)	*P* value
Medicaid	9.31 (6.86, 11.75)	<.001	10.67 (8.15, 13.19)	<.001
Private Coverage	-2.48 (-4.96, -0.00)	0.05	-3.50 (-5.89, -1.12)	0.006
Dual Medicaid and Private	0.95 (-0.94, 2.84)	0.31	0.66 (-1.66, 2.99)	0.56
Uninsurance	-5.87 (-8.81, -2.93)	<.001	-6.50 (-9.26, -3.75)	<.001

Supplemental Table 7. Sensitivity Analysis: Donut Regression Discontinuity

This sensitivity analysis presents main models excluding births that occurred in January-March 2020 to omit any births that may have occurred prior to the March 18, 2020 Families First Coronavirus Response Act Medicaid provisions. Individuals whose births occurred in January-March should have maintained pregnancy-related Medicaid coverage by the Medicaid policy changes, as it is within the window of pregnancy-related Medicaid eligibility that lasts through the end of the month in which the 60 days postpartum period occurs. However, it is possible that some individuals may have transitioned away from Medicaid coverage prior to March 18, 2020 in anticipation of the pregnancy-related Medicaid eligibility ending. As a result, we conducted a donut regression discontinuity approach omitting births that occurred in January-March 2020 and considering the discontinuity to occur at April 2020 rather than January 2020 as in main models. Using a donut regression discontinuity design, we found results that were similar to main models in regard to effect size and statistical significance for postpartum Medicaid, private coverage, and uninsurance in adjusted models. However, we found significant increases in dual Medicaid and private coverage in our donut regression discontinuity design models, which were not observed in main models. As a result, estimates of the association of extended postpartum Medicaid eligibility with dual coverage enrollment appear to be sensitive to omitting births that occurred in January-March 2020.

Outcome	Unadjusted Discontinuity at 4/20 (95% CI)	*P* value	Adjusted Discontinuity at 4/20 (95% CI)	*P* value
Medicaid	10.76 (8.10, 13.42)	<.001	11.66 (9.87, 13.44)	<.001
Private Coverage	-2.23 (-4.45, -0.02)	0.05	-3.01 (-4.69, -1.32)	0.001
Dual Medicaid and Private	1.89 (0.17, 3.60)	0.03	1.53 (0.15, 2.91)	0.03
Uninsurance	-6.64 (-8.38, -4.90)	<.001	-7.12 (-8.41, -5.83)	<.001

Supplemental Table 8. Sensitivity Analysis: Data-Driven Narrow Bandwidth Approach

In our primary specification, we included the full sample for a global parametric regression discontinuity design. In this sensitivity analysis, we used an alternative method that includes observations only within a specific narrow bandwidth around the cutoff, employing a data-driven approach through the “rdrobust” package in Stata to select the optimal bandwidth. From this package, we report the bias-corrected regression discontinuity estimates with robust variance estimators. Under this approach, the data-driven narrow bandwidth for the regression discontinuity estimate varied by outcome, ranging from 6.526 to 8.405 months around the cutoff in adjusted models. In adjusted models, we found results similar to the main models for significant increases in postpartum Medicaid and no statistically significant changes in dual coverage. However, using the narrow bandwidth approach produces meaningfully larger estimates for decreases in postpartum uninsurance during COVID-19 continuous coverage provisions and finds no evidence of a statistically significant decrease in postpartum private coverage, which were observed in main models. As a result, our estimates in the main models for postpartum private coverage appear to be sensitive to the bandwidth specification.

Outcome	Unadjusted Discontinuity at 2020 (95% CI)	*P* value	Adjusted Discontinuity at 2020 (95% CI)	*P* value
Medicaid	4.76 (-0.14, 4.26)	0.07	11.18 (10.94, 12.97)	<.001
Private Coverage	-1.43 (-3.45, 0.82)	0.23	1.50 (-0.33, 1.39)	0.23
Dual Medicaid and Private	1.72 (0.00, 3.43)	0.05	0.68 (-1.69, 0.51)	0.30
Uninsurance	-3.57 (-3.89, -0.35)	0.02	-11.88 (-14.43, -11.42)	<.001

Supplemental Table 9. Sensitivity Analysis: Population of Medicaid-Paid Deliveries

This sensitivity analysis uses an alternative study sample of respondents who had Medicaid at delivery as reported on the birth certificate files, which are linked to the PRAMS data. This sample could yield varying outcomes from our main models for two reasons. First, this delivery coverage variable is from the birth certificate files rather than from self-report in PRAMS like prenatal and postpartum coverage. As a result, some changes in postpartum coverage from delivery coverage may be attributable to differences in the data source and measurement rather than true insurance changes. Secondly, individuals with Medicaid coverage at delivery may differ from individuals with prenatal Medicaid as some birthing people get emergency Medicaid just for delivery care. Emergency Medicaid provides temporary Medicaid coverage for emergency medical care, including labor and delivery, for immigrants who meet Medicaid eligibility standards but do not qualify due to their immigration status.[4] As a result, Medicaid recipients covered through this program are only covered by Medicaid during the delivery hospitalization, and therefore do not have Medicaid coverage beforehand in the prenatal period or afterwards in the postpartum period. However, despite these two factors potentially driving differences in results, we found results that were similar to main models in regards to effect sizes and statistical significance.

Outcome	Baseline Coverage Rates, 2018-19	Unadjusted Discontinuity at 2020 (95% CI)	*P* value	Adjusted Discontinuity at 2020 (95% CI)	*P* value
Medicaid	70.30	8.90 (6.20, 11.59)	<.001	10.54 (8.77, 12.30)	<.001
Private Coverage	15.25	-1.81 (-3.62, 0.00)	0.05	-2.20 (-3.63, -0.78)	0.003
Dual Medicaid and Private	4.09	0.01 (-1.27, 1.28)	0.99	-0.27 (-1.36, 0.83)	0.63
Uninsurance	18.55	-7.08 (-9.12, -5.04)	<.001	-8.60 (-10.26, -6.94)	<.001

Appendix References

1. Ding D, Sommers BD, Glied SA. Unwinding And The Medicaid Undercount: Millions Enrolled In Medicaid During The Pandemic Thought They Were Uninsured: Study examines the expiration of the Medicaid continuous coverage provision and improving the accuracy of Medicaid enrollment assessments. *Health Affairs*. 2024;43(5):725-731. doi:10.1377/hlthaff.2023.01069
2. Admon LK, Daw JR, Interrante JD, Ibrahim BB, Millette MJ, Kozhimannil KB. Rural and Urban Differences in Insurance Coverage at Prepregnancy, Birth, and Postpartum. *Obstetrics & Gynecology*. Published online February 2, 2023. doi:10.1097/AOG.0000000000005081
3. Hausman C, Rapson D. *Regression Discontinuity in Time: Considerations for Empirical Applications*. National Bureau of Economic Research; 2017:w23602. doi:10.3386/w23602
4. Centers for Medicare & Medicaid Services. Health Coverage Options for Immigrants. Enrollment Resources - Materials for Special Populations. July 2022. https://www.cms.gov/marketplace/technical-assistance-resources/health-coverage-options-immigrants.pdf
